# Surgical risk stratification and outcome analysis of Tenckhoff catheter implantations in paediatric patients: a single-centre experience

**DOI:** 10.1007/s00431-025-06006-x

**Published:** 2025-02-04

**Authors:** Michail Aftzoglou, Katerina Dadouli, Gwendolyn Eich, Konrad Reinshagen, Christian Tomuschat

**Affiliations:** 1 Department of Paediatric Surgery, University Medical Centre Hamburg-Eppendorf (UKE), 20246 Hamburg, Germany; 2 Laboratory of Hygiene and Epidemiology, Faculty of Medicine, University of Thessaly, 41222 Larissa, Greece; 3 Department of Paediatrics, University Medical Centre Hamburg-Eppendorf (UKE), 20246 Hamburg, Germany

**Keywords:** Surgical outcome, Tenckhoff catheter, Peritoneal dialysis, Children, High-risk patients

## Abstract

This study aimed to assess the risk factors for complications post-Tenckhoff catheter implantation in paediatric patients. All records of children who underwent the procedure from 2002 to 2022 at the University Medical Centre of Hamburg were analyzed. The demographic and anthropometric characteristics were scrutinized, with particular attention given to complications such as catheter leakage, occlusions, and peritonitis. Univariate and multivariate analyses were employed to determine the hazard ratios for complications. In total, 299 implantations were performed in 116 females and 130 males; 67% were under 5 years old, and 85% had renal disease. Fifty-one percent needed an acute catheter insertion, 26% of the patient‘s required revision, and 24% of the patients experienced peritonitis. Neonates had a 22% mortality rate, and infants had a 37% reimplantation rate. Compared with the other subgroups, a glomerular filtration rate of 15–29 ml/kg/1.7 3m^2^ was associated with a 10.7-fold higher risk of peritonitis. Male patients had a threefold greater chance of reimplantation or inguinal hernia. Omentectomy revealed no increased risk of peritonitis, and only two catheter occlusions were observed. Patients with inguinal hernias or gastric tubes had higher odds of complications (HR = 3.60, *p* = 0.003 and OR = 2.47, *p* = 0.014). Neither the implantation side nor acute insertion was correlated with complications.

*Conclusion*: Infants, male patients with kidney disease, prolonged use of catheter, and those with a GFR of 15–29 ml/kg/1.73 m^2^ were at increased risk. The presence of peritonitis, inguinal hernia, and gastric tubes were adverse prognostic factors.

**Table:** 

**What is Known:** • *Omentectomy can serve as a protective factor and correlates with a lower incidence of catheter malfunction.* • *The insertion of gastric tubes is commonly associated with complications but has lower risk of complications if inserted prior to the initiation of peritoneal dialysis.*
**What is New:** • *Males have been found to have a 2.14 hazard ratio for revision in both acute and chronic peritoneal dialysis groups.* • *The incidence of revisions seems to be greater in the first 36 months, while the incidence of peritonitis rises by 7% for each additional year of age.*

**Supplementary Information:**

The online version contains supplementary material available at 10.1007/s00431-025-06006-x.

## Introduction

In paediatric populations suffering from renal failure, peritoneal dialysis (PD) has emerged as the therapeutic modality of choice [1, 2]. It is also uniquely suitable for use in hemodynamically unstable patients and neonates, allowing the maintenance of vascular access and avoidance of a coagulation treatment [3]. A significant advantage of PD is its superior preservation of residual renal function, which means that the patients have less restrictive dietary requirements and possibly better survival in the first few years [4, 5]. However, certain contraindications for PD include the presence of abdominal wall or diaphragm defects, bladder exstrophy, or peritoneal membrane failure [6]. These conditions necessitate careful consideration regarding the selection of the most appropriate treatment [4, 6]. Aggressive nutritional support of patients during PD is imperative to avoid caloric deficit and to overcome the hurdle of anorexia, especially in infants [7, 8]. The use of nasogastric or gastric tubes, as well as the incidence of hernia, does not inherently lead to complications but necessitates a patient-specific safety evaluation [8]. Currently, the most prevalent catheter employed is the silicon Dacron with cuffs and curved-end Tenckhoff catheter (TC) [9]. The merits of this catheter include its pliability, subcutaneous positioning, and stabilization via the cuffs, diminishing complications such as dislodgement, leakage, and infections [9, 10]. Despite the strides that have been made in surgery, catheter-related complications remain common in paediatric patients undergoing PD [11, 12]. To increase the quality of patient care, standardized procedural guidelines for TC insertion have now been made public [13]. The scarcity of published research on catheter-related complications from smaller-scale studies the past 10 years has yielded conflicting results [4, 9, 12, 13]. The aim of this retrospective study is to identify the factors influencing these complications and stratify patients according to the risks associated with PD.

## Material and methods

This was a single-centre retrospective analysis examining the medical records of paediatric patients after the implantation of a curved-end TC for PD at the University Medical Centre of Hamburg-Eppendorf, Germany (UMC-HE), between 2002 and 2022. Patient data were extracted from our institutional operational database system (MyMedis, Getinge IT Solutions, Germany, copyright 1994–2024). This study included children less than 18 years old at the time of TC implantation. Patients who were adults had other catheter types or whose records were incomplete were excluded from this study’s cohort.

### Patient’s medical records

The patients’ demographic and anthropometric characteristics were collected, including their original kidney diagnosis and the specifics of their serum biochemistry, on the day of the catheter’s implantation. The eGFR was calculated via the Schwartz formula [16]. Additional data gleaned from medical records included information regarding the indication for PD and kidney transplantation, death, the duration of PD, partial omentectomy, and the side of implantation. The practitioner’s level of experience with this procedure was stratified as high (> 10 years), moderate (5–10 years), or low (< 5 years). A specific focus was given to instances of peritonitis and catheter malfunction. The occurrence of an inguinal hernia, history of previous abdominal surgeries, and feeding gastric tube insertion were also documented.

### Definitions

Catheter use was measured from initiation, as recorded in the PD protocol, to cessation marked by either catheter removal or revision. Acute PD cases involved acute kidney injury (AKI) following events such as heart surgery, metabolic diseases, polytrauma, or haemolytic uraemic syndrome. Catheter dysfunction included any obstruction or dislocation noted in the operation records. Leakage was clinically identified by visually detecting fluid escaping from the wound during dialysis, as documented in the patient files. Catheter reimplantation referred to any manipulation under general anaesthesia during the usage phase. Peritonitis was diagnosed according to the standard criteria of the International Paediatric Peritoneal Dialysis Network [1, 2]. The WHO classification was followed for the subcategories of preterm birth and age groups.

### Outcome measures

The primary outcome measures included complications associated with TC implantation, namely, leakage, dislocation, occlusion, and peritonitis. The secondary outcome measures included the prevalence of inguinal hernias and complications resulting from the insertion of a feeding gastric tube or other abdominal surgeries.

### Surgical treatment

At UMC-HE cardiac, transplant surgeons or paediatric surgeons placed the catheters. A Tenckhoff catheter with a curved end (Covidien; Mansfield, MA, USA) was inserted laparoscopically or via a mini laparotomy. After considering the projected distance from the umbilicus to the symphysis pubis, the selection of the catheter size and start of usage was decided collaboratively by the surgical and the nephrology teams. Antibiotics were administered after the nephrological assessment. The catheter was placed according to the guidelines of the European Paediatric Peritoneal Dialysis Working Group [1, 2]. From 2013 onwards, partial omentectomy became a standard practice. Catheter integrity was tested by injecting sterile NaCl 0.9% solution (30 ml/kg body weight) [1, 2], which was passively drained at the end of the operation.

### Statistical analysis

Data management was performed with SPSS version 22 (SPSS Inc., Chicago, IL, USA/IBM Armonk, NY, USA), and statistical analyses were performed with R language (version 4.2.2) (R Core Team: R: A Language and Environment for Statistical Computing Vienna, Austria: Foundation for Statistical Computing. Available from: http://www.R-project.org/). Ggplot2, Table 1, survminer, and survival packages were used. Categorical variables are presented as frequencies and percentages, whereas continuous variables are summarized as medians and interquartile ranges (IQRs). Categorical data were analyzed using the chi-square test. A Poisson regression model was used to compare the incidence of complications. A Kaplan–Meier estimate with a log-rank test was used to compare the time to first complication (revisions, peritonitis, and hernias) between the understudy variables/risk factors of interest. Hazard ratios (HRs) estimated with univariate Cox regression models were applied to estimate the effect of the parameters of interest on the time to first complication. The multivariate models were adjusted to the most important risk factors reported in the literature [5, 6]. Statistical significance was set at a two-sided *p*-value of 0.050.

**Table 1:** Patients’ characteristics

Descriptive analysis	One operation	Two operations	Three operations	Five operations	Overall
	(*N* = 246)	(*N* = 40)	(*N* = 12)	(*N* = 1)	(*N* = 299)
Age group					
Newborn	53 (21.5%)	8 (20.0%)	0 (0.0%)	0 (0.0%)	61 (20.4%)
Infant	32 (13.0%)	11 (27.5%)	8 (66.7%)	0 (0.0%)	51 (17.1%)
Toddler	80 (32.5%)	8 (20.0%)	1 (8.3%)	1 (100.0%)	90 (30.1%)
School child	44 (17.9%)	10 (25.0%)	2 (16.7%)	0 (0.0%)	56 (18.7%)
Post adolescent, adolescent, adults	37 (15.0%)	3 (7.5%)	1 (8.3%)	0 (0.0%)	41 (13.7%)
BMI					
Less than the 5th percentile	50 (20.3%)	5 (12.5%)	0 (0.0%)	0 (0.0%)	55 (18.4%)
5th percentile to less than the 85th percentile	102 (41.5%)	10 (25.0%)	3 (25.0%)	0 (0.0%)	115 (38.5%)
85th percentile to less than the 95th percentile	12 (4.9%)	1 (2.5%)	0 (0.0%)	0 (0.0%)	13 (4.3%)
95th percentile or greater	13 (5.3%)	1 (2.5%)	2 (16.7%)	0 (0.0%)	16 (5.4%)
Missing	69 (28.0%)	23 (57.5%)	7 (58.3%)	1 (100%)	100 (33.4%)
Gestations age cat					
Extremely PT (< 28)	3 (1.2%)	2 (5.0%)	1 (8.3%)	0 (0.0%)	6 (2.0%)
Very PT (> 28– < 32)	10 (4.1%)	5 (12.5%)	2 (16.7%)	0 (0.0%)	17 (5.7%)
Moderately PT (> 32– < 34)	11 (4.5%)	2 (5.0%)	1 (8.3%)	1 (100.0%)	15 (5.0%)
Late PT (> 34– < 37)	30 (12.2%)	6 (15.0%)	2 (16.7%)	0 (0.0%)	38 (12.7%)
Full term (> 37)	45 (18.3%)	8 (20.0%)	3 (25.0%)	0 (0.0%)	56 (18.7%)
Missing	147 (59.8%)	17 (42.5%)	3 (25.0%)	0 (0%)	167 (55.9%)
Creatinine					
Median [IQR]	3.90 [3.56]	4.00 [3.30]	3.40 [1.67]	4.43 [0]	3.90 [3.55]
Diseases group					
Heart disease	29 (11.8%)	5 (12.5%)	0 (0.0%)	0 (0.0%)	34 (11.4%)
Kidney disease	209 (85.0%)	35 (87.5%)	12 (100.0%)	1 (100.0%)	257 (86.0%)
Other	8 (3.3%)	0 (0.0%)	0 (0.0%)	0 (0.0%)	8 (2.7%)
Omentectomy					
No	180 (73.2%)	31 (77.5%)	8 (66.7%)	1 (100.0%)	220 (73.6%)
Yes	56 (22.8%)	8 (20.0%)	4 (33.3%)	0 (0.0%)	68 (22.7%)
Missing	10 (4.1%)	1 (2.5%)	0 (0%)	0 (0%)	11 (3.7%)
Experience					
Low experience	41 (16.7%)	5 (12.5%)	1 (8.3%)	1 (100.0%)	48 (16.1%)
Moderate experience	64 (26.0%)	10 (25.0%)	1 (8.3%)	0 (0.0%)	75 (25.1%)
High experience	96 (39.0%)	18 (45.0%)	9 (75.0%)	0 (0.0%)	123 (41.1%)
Missing	45 (18.3%)	7 (17.5%)	1 (8.3%)	0 (0%)	53 (17.7%)
Side of implantation					
Left	122 (49.6%)	18 (45.0%)	4 (33.3%)	0 (0.0%)	144 (48.2%)
Right	71 (28.9%)	19 (47.5%)	7 (58.3%)	1 (100.0%)	98 (32.8%)
Other	5 (2.0%)	0 (0.0%)	0 (0.0%)	0 (0.0%)	5 (1.7%)
Missing	48 (19.5%)	3 (7.5%)	1 (8.3%)	0 (0%)	52 (17.4%)

## Results

### Participant demographics

From the a total of 279 patients, only 246 were included, as 12 patients in the operation note were missing and 17 underwent PD with a different type of catheter. From the remaining 246 patients (116 females, 130 males), 299 underwent TC implantations (Table 1). In total, 51% of them had an acute indication. The age group breakdown was 53 newborns (0–28 days), 32 infants (29 days to 12 months), 80 toddlers (1–3 years), 44 school-aged children (4–10 years), and 37 adolescents (10–19 years). Patients required one to more than three catheter reimplantations (40 [16%] needed two, 12 [4.9%] needed three, and 1 [0.4%] needed five). Diseases were categorized as heart disease—mostly congenital heart vessel transposition and septum defects (29 patients, 11%)—kidney disease (209 patients, 85%), or both. ECMO was utilized in 17 patients during congenital cardiac malformation surgery and a PD catheter was indicated for AKI management. Additionally, 7 patients (5 with metabolic disease [2%] and 2 with short bowel syndrome and polytrauma) underwent TC implantation. Omentectomy was performed in 56 patients (23%). The implantation sites varied: left lower abdominal quadrant (144, 48%), right lower quadrant (98, 32%), middle abdominal line (5, 1.8%), and 52 (17%) were unrecorded (Tables 1 and 2).

**Table 2:** Patients’ characteristics

Descriptive analysis	One operation (*N* = 246)	Two operations (*N* = 40)	Three operations (*N* = 12)	Five operations (*N* = 1)	
Revision					
No	180 (73.2%)	29 (72.5%)	9 (75.0%)	1 (100.0%)	219 (73.2%)
Yes	65 (26.4%)	11 (27.5%)	3 (25.0%)	0 (0.0%)	79 (26.4%)
Missing	1 (0.4%)	0 (0%)	0 (0%)	0 (0%)	1 (0.3%)
Number of peritonitis					
0	183 (74.4%)	26 (65.0%)	9 (75.0%)	0 (0.0%)	218 (72.9%)
1	43 (17.5%)	8 (20.0%)	2 (16.7%)	0 (0.0%)	53 (17.7%)
2	11 (4.5%)	5 (12.5%)	0 (0.0%)	1 (100.0%)	17 (5.7%)
3	3 (1.2%)	0 (0.0%)	1 (8.3%)	0 (0.0%)	4 (1.3%)
4	5 (2.0%)	0 (0.0%)	0 (0.0%)	0 (0.0%)	5 (1.7%)
Missing	1 (0.4%)	1 (2.5%)	0 (0%)	0 (0%)	2 (0.7%)
First peritonitis					
No	183 (74.4%)	26 (65.0%)	9 (75.0%)	0 (0.0%)	218 (72.9%)
Yes	62 (25.2%)	13 (32.5%)	3 (25.0%)	1 (100.0%)	79 (26.4%)
Missing	1 (0.4%)	1 (2.5%)	0 (0%)	0 (0%)	2 (0.7%)
Time to first peritonitis (months)					
Mean (SD)	78.6 (71.8)	69.6 (74.1)	63.6 (64.3)	NA	76.5 (71.7)
Median [IQR]	55.1 [127]	31.0 [104]	48.6 [81.3]	0 [0]	53.6 [128]
Second peritonitis					
No	225 (91.5%)	34 (85.0%)	11 (91.7%)	1 (100.0%)	271 (90.6%)
Yes	20 (8.1%)	5 (12.5%)	1 (8.3%)	0 (0.0%)	26 (8.7%)
Missing	1 (0.4%)	1 (2.5%)	0 (0%)	0 (0%)	2 (0.7%)
Time between first and second peritonitis (months)					
Median [IQR]	2.43 [5.27]	1.07 [5.09]	26.6 [0]	NA	2.23 [6.33]
Third peritonitis					
No	236 (95.9%)	39 (97.5%)	11 (91.7%)	1 (100.0%)	287 (96.0%)
Yes	9 (3.7%)	0 (0.0%)	1 (8.3%)	0 (0.0%)	10 (3.3%)
Missing	1 (0.4%)	1 (2.5%)	0 (0%)	0 (0%)	2 (0.7%)
Time between second and third peritonitis (months)					
Median [IQR]	1.17 [1.58]	0 [0]	0.300 [0]	NA	0.917 [1.92]
Fourth peritonitis					
No	239 (97.2%)	39 (97.5%)	12 (100.0%)	1 (100.0%)	291 (97.3%)
Yes	6 (2.4%)	0 (0.0%)	0 (0.0%)	0 (0.0%)	6 (2.0%)
Missing	1 (0.4%)	1 (2.5%)	0 (0%)	0 (0%)	2 (0.7%)
Time fourth peritonitis (months)					
Median [IQR]	0 [0]	0 [0]	0 [0]	0 [0]	0 [0]
Hernia inguinal					
No	213 (86.6%)	31 (77.5%)	7 (58.3%)	1 (100.0%)	252 (84.3%)
Hernia	30 (12.2%)	7 (17.5%)	4 (33.3%)	0 (0.0%)	41 (13.7%)
Hydrocele	3 (1.2%)	2 (5.0%)	1 (8.3%)	0 (0.0%)	6 (2.0%)
Gastric tube					
No	209 (85.0%)	29 (72.5%)	7 (58.3%)	0 (0.0%)	245 (81.9%)
Yes	37 (15.0%)	11 (27.5%)	5 (41.7%)	1 (100.0%)	54 (18.1%)
Death					
No	204 (82.9%)	30 (75.0%)	8 (66.7%)	1 (100.0%)	243 (81.3%)
Yes	42 (17.1%)	10 (25.0%)	4 (33.3%)	0 (0.0%)	56 (18.7%)

### Survival analysis

Fifty-six deaths occurred within 61 months. The mortality rate increased with the number of operations, rising from 17.1% after one operation to 33.3% after three operations. No deaths were observed following five operations. Overall, 18.7% of patients experienced mortality across all groups. The median catheter survival time was 0.6 years. Male patients and patients with kidney disease, aBMI > the 95th percentile (HR = 3.75, *p* = 0.013), and a GFR ranging from 15 to 29 (HR = 4.11, *p* = 0.010) were associated with significantly worse survival. Patients with non-cardiac/non-renal causes, such as trauma or metabolic disease, had a relatively greater risk of mortality (aHR = 5.99, *p* = 0.002). The occurrence of peritonitis (aHR = 0.81, *p* = 0.607) and hernias (aHR = 0.22, *p* = 0.138) was not associated with decreased survival. Prematurity negatively influenced survival in those < 12 months (aHR = 16.22, *p* = 0.014) (Table 3). Surgical experience (high vs. low, HR = 2.72, *p* = 0.139; moderate vs. low, HR = 2.58, *p* = 0.106), the use of a gastric tube (aHR = 1.23, *p* = 0.631), and number of operations (2 vs 1, aHR = 2.55, *p* = 0.059; 3 vs 1, aHR = 6.77, *p* = 0.062) marginally influenced survival. Children < 12 months with right-sided TC implantation had greater survival (aHR = 0.16, *p* = 0.037).

**Table 3:** Overall survival (total sample)

Factor		*N* (%)	HR with 95% CI	aHR with 95% CI
Sex	Female	116 (47.2)	Ref	Ref
	Male	130 (52.8)	1.80 (0.94–3.42, *p* = 0.076)	0.91 (0.38–2.13, *p* = 0.820)
Age group	Newborn	53 (21.5)	Ref	Ref
	Infant	32 (13.0)	1.37 (0.66–2.81, *p* = 0.396)	1.60 (0.71–3.63, *p* = 0.259)
	Toddler	80 (32.5)	0.23 (0.10–0.56, *p* = 0.001)	0.65 (0.24–1.75, *p* = 0.393)
	School child	44 (17.9)	0.06 (0.01–0.43, *p* = 0.005)	- (*p* = 0.998)
	Post adolescent, adolescent, adults	37 (15.0)	0.22 (0.06–0.74, *p* = 0.015)	- (*p* = 0.998)
Diseases group	Heart disease	29 (11.8)	Ref	Ref
	Kidney disease	209 (85.0)	0.17 (0.09–0.34, *p* < 0.001)	0.44 (0.19–1.00, *p* = 0.051)
	Other*	8 (3.3)	1.90 (0.68–5.29, *p* = 0.219)	5.99 (1.97–18.21, *p* = 0.002)
Omentectomy	No	180 (76.3)	Ref	Ref
	Yes	56 (23.7)	0.85 (0.39–1.85, *p* = 0.683)	0.33 (0.08–1.44, *p* = 0.140)
Side of implantation	Left	122 (61.6)	Ref	Ref
	Right	71 (35.9)	0.88 (0.44–1.77, *p* = 0.728)	0.56 (0.22–1.42, *p* = 0.219)
	Other	5 (2.5)	2.06 (0.49–8.72, *p* = 0.326)	4.74 (1.08–20.77, *p* = 0.039)
Experience	Low experience	41 (20.4)	Ref	Ref
	Moderate experience	64 (31.8)	2.58 (0.73–9.08, *p* = 0.139)	-
	High experience	96 (47.8)	2.72 (0.81–9.16, *p* = 0.106)	-
BMI	5th percentile to less than the 85th percentile	102 (57.6)	Ref	Ref
	Less than the 5th percentile	50 (28.2)	1.37 (0.56–3.36, *p* = 0.487)	1.05 (0.44–2.48, *p* = 0.916)
	85th percentile to less than the 95th percentile	12 (6.8)	- (*p* = 0.997)	- (*p* = 0.998)
	95th percentile or greater	13 (7.3)	3.75 (1.32–10.66, *p* = 0.013)	1.82 (0.66–5.02, *p* = 0.245)
Creatinine	Mean (SD)	5.1 (8.1)	0.63 (0.53–0.76, *p* < 0.001)	0.84 (0.64–1.10, *p* = 0.200)
GFR	< 15	150 (82.9)	Ref	Ref
	90	2 (1.1)	- (*p* = 0.997)	- (*p* = 1.000)
	60–89	2 (1.1)	8.69 (1.15–65.91, *p* = 0.036)	1.29 (0.11–14.88, *p* = 0.839)
	30–59	8 (4.4)	4.11 (1.40–12.04, *p* = 0.010)	0.83 (0.16–4.48, *p* = 0.832)
	15–29	19 (10.5)	1.33 (0.40–4.49, *p* = 0.642)	0.61 (0.13–2.91, *p* = 0.534)
Number of peritonitis	Mean (SD)	0.4 (0.8)	0.70 (0.42–1.18, *p* = 0.182)	0.81 (0.36–1.82, *p* = 0.607)
Revision	No	180 (73.5)	Ref	Ref
	Yes	65 (26.5)	1.12 (0.57–2.19, *p* = 0.742)	0.76 (0.31–1.84, *p* = 0.547)
Hernia	No	213 (86.6)	Ref	Ref
	Yes	33 (13.4)	0.30 (0.07–1.23, *p* = 0.093)	0.22 (0.03–1.63, *p* = 0.138)
No of operations	1	204 (82.9)	Ref	Ref
	2	30 (12.2)	1.36 (0.57–3.26, *p* = 0.491)	2.55 (0.97–6.74, *p* = 0.059)
	3	11 (4.5)	2.40 (0.85–6.79, *p* = 0.100)	6.77 (0.91–50.53, *p* = 0.062)
	5	1 (0.4)	- (*p* = 0.996)	- (*p* = 1.000)
Gastric tube	No	209 (85.0)	Ref	Ref
	Yes	37 (15.0)	1.80 (0.88–3.67, *p* = 0.107)	1.23 (0.52–2.91, *p* = 0.631)
Hernia	No	65 (76.5)	Ref	Ref
	Yes	20 (23.5)	0.17 (0.04–0.73, *p* = 0.017)	-
No of operations	1	61 (71.8)	Ref	Ref
	2	16 (18.8)	1.23 (0.49–3.08, *p* = 0.653)	-
	3	7 (8.2)	1.66 (0.57–4.86, *p* = 0.356)	-
	5	1 (1.2)	- (*p* = 0.998)	-
Gastric tube	No	62 (72.9)	Ref	Ref
	Yes	23 (27.1)	0.44 (0.17–1.15, *p* = 0.092)	0.33 (0.06–1.87, *p* = 0.211)

*Bowel disease, metabolic disease, trauma (+ 1pts kidney and heart disease)

*HR*, hazard ratio; *aHR*, adjusted hazard ratio; *CI*, confidence interval

### Analysis of revisions

The analysis of seventy-nine revisions showed that males with kidney disease were more likely to undergo revision (HR = 2.05, *p* = 0.003), while older patients were less likely to undergo revision (Table 4). The risk of revision was independent of implantation side (right vs. left aHR = 1.58, *p* = 0.210; right vs. other aHR = 6.94, *p* = 0.035) or omentectomy (aHR = 1.04, *p* = 0.939). Two patients who underwent omentectomy experienced catheter obstruction. Patients with kidney disease had a higher reimplantation risk. Acute PD induction was associated with a lower risk of revision (aHR = 0.35, *p* = 0.055). The incidence of revision was higher in the first 36 months and declined thereafter. Greater surgical experience was associated with a greater risk of revision (aHR = 2.19, *p* = 0.240). The common causes of revision were leakage (32%), dislocation (18%), obstruction (15%), and other dysfunctions (5%).

**Table 4:**
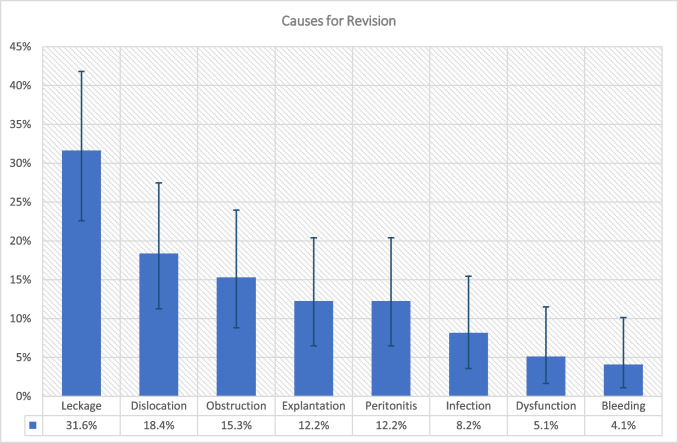
Revision, time to event analysis (all participants)

### Incidence of peritonitis

Univariate analysis suggested that males with kidney disease had a greater risk of a first peritonitis episode (HR = 1.36, *p* = 0.260), although these findings was not confirmed in multivariate analysis (aHR = 1.08, *p* = 0.840) (Table 5). The presence of gastric tubes was a significant predictor of peritonitis (aHR = 2.89, *p* = 0.021). Among infants, moderate preterm newborns and those with GFR 15–29 were associated with an increased risk of peritonitis (HR = 15.44, *p* = 0.003 and HR = 10.7, *p* = 0.025). Acute PD induction was associated with a decreased risk of peritonitis (aHR = 0.09, *p* < 0.001). The incidence of peritonitis increased by 7% with each additional year of age (aIRR = 1.07, *p* = 0.045) (Table 5). The overall peritonitis incidence rate was 0.06 per patient per year. The risk of peritonitis was not increased in the subgroup of patients who underwent omentectomy (RR = 0.74, *p* = 0.458). The Poisson regression linked patients with peritonitis within 3 months post-implantation to a twofold higher incidence of recurring peritonitis, particularly in kidney disease patients (aIRR = 2.53, *p* = 0.021).

**Table 5:** Peritonitis, time to event analysis (all participants)

		All	HR with 95% CI	aHR with 95% CI
Sex	Female	116 (47.2)	Ref	Ref
	Male	130 (52.8)	1.28 (0.77–2.12, *p* = 0.336)	1.17 (0.60–2.30, *p* = 0.645)
Age group	Newborn	53 (21.5)	Ref	Ref
	Infant	32 (13.0)	0.96 (0.40–2.29, *p* = 0.930)	1.28 (0.38–4.24, *p* = 0.690)
	Toddler	80 (32.5)	0.63 (0.30–1.32, *p* = 0.223)	0.52 (0.17–1.58, *p* = 0.249)
	School child	44 (17.9)	1.10 (0.52–2.35, *p* = 0.800)	0.82 (0.27–2.51, *p* = 0.730)
	Post adolescent, adolescent, adults	37 (15.0)	1.33 (0.62–2.83, *p* = 0.461)	0.63 (0.18–2.20, *p* = 0.470)
Diseases	Heart disease	29 (11.8)	Ref	Ref
	Kidney disease	209 (85.0)	1.60 (0.64–4.00, *p* = 0.312)	1.24 (0.29–5.27, *p* = 0.772)
	Other	8 (3.3)	0.63 (0.07–5.35, *p* = 0.668)	- (*p* = 0.997)
BMI	5th percentile to less than the 85th percentile	102 (57.6)	Ref	Ref
	Less than the 5th percentile	50 (28.2)	0.82 (0.41–1.66, *p* = 0.586)	0.63 (0.25–1.56, *p* = 0.318)
	85th percentile to less than the 95th percentile	12 (6.8)	0.81 (0.25–2.69, *p* = 0.736)	1.02 (0.29–3.53, *p* = 0.981)
	95th percentile or greater	13 (7.3)	1.13 (0.40–3.24, *p* = 0.815)	1.25 (0.30–5.16, *p* = 0.755)
Creatinine	Mean (SD)	5.1 (8.1)	1.02 (0.99–1.05, *p* = 0.186)	1.19 (1.06–1.34, *p* = 0.002)
GFR	< 15	150 (82.9)	Ref	Ref
	90	2 (1.1)	- (*p* = 0.997)	- (*p* = 0.998)
	60–89	2 (1.1)	- (*p* = 0.997)	- (*p* = 0.998)
	30–59	8 (4.4)	0.87 (0.21–3.60, *p* = 0.850)	2.08 (0.33–13.08, *p* = 0.436)
	15–29	19 (10.5)	1.02 (0.40–2.59, *p* = 0.961)	2.40 (0.61–9.40, *p* = 0.208)
Gastric tube	No	209 (85.0)	Ref	Ref
	Yes	37 (15.0)	2.91 (1.69–5.00, *p* < 0.001)	2.89 (1.17–7.10, *p* = 0.021)

*Bowel disease, metabolic disease, trauma (+ 1pts kidney and heart disease)

*HR*, hazard ratio; *aHR*, adjusted hazard ratio; *CI*, confidence interval

### Inguinal hernia incidence

The incidence of inguinal hernia was 14%, with an additional 2% of males exhibiting hydrocele (Table 2). The risk was greater in newborns and children < 3 years of age. Kidney disease and a higher GFR were associated with a greater risk of hernia (HR = 8.85, *p* = 0.088 and HR = 12.18, *p* = 0.054). A similar incidence of inguinal hernia was reported across the BMI and surgeon’s experience subgroup. Younger age at PD initiation was associated with an increased hernia risk, with newborns having the highest risk.

## Discussion

Despite technological advancements and improved safety in PD, mortality rates for children requiring renal replacement therapy remain 30 times greater than those of healthy peers [1, 13, 14]. To improve treatment quality and long-term survival outcomes, identifying risk of complication is essential [15]. In this study, patients with acute kidney injury (AKI) and end-stage renal disease received PD. UMC-HE serves as a regional centre for managing rare heart and kidney diseases and facilitates transplants across northern Germany. Fragile newborns and infants comprised one-third of the cohort, with 51% requiring acute PD. Following the haemolytic uremic syndrome epidemic in May 2011, the number of toddlers and school-age children requiring PD increased, highlighting novel risk factors specific to this age group [17, 18].

Reference studies report a mortality range of 7 to 32%, aligning with the 23% observed in this study, or even higher among infants or children with AKI [19]. The International Society for Peritoneal Dialysis recommends TC implantation at least 2 weeks before dialysis to facilitate proper healing [1, 20]. However, acute PD initiation and catheter usage are often linked with complications. This study’s population demonstrated a lower risk of catheter revision, likely due to the shorter PD duration of compared to chronic kidney patients. The cohort’s median survival was 83 months, while median catheter survival was 0.6 years, which is similar to data from retrospective studies conducted in the USA and the Netherlands [21–23]. Newborns, particularly, moderate preterm infants, exhibited a 4.75-fold higher risk of complications [19, 21]. Factors such as thin abdominal walls, poor nutritional status, and challenging vascular access necessitated earlier TC use, increasing their risk. Additionally, children requiring PD for non-cardiac, non-renal diseases demonstrated significantly decreased physiological reserves resulting in no survivors within this group.

Modern TCs are single- or double-cuffed catheters with curled ends and a median or paramedian exit site [9]. While double-cuffed catheters are preferred [8], TC implantation poses technical challenges and is associated with higher complication rates in infants weighing less than 10 kg [22]. This study observed an overall revision rate of 26%, predominantly due to dislocations and infections, with a greater risk of revisions in chronic PD patient. Radtke et al. combined data from UMC-HE and Charité children’s department reporting similar outcomes.

Revision rates vary widely, while Phan et al. reported rates up to 49% in the USA and the Italian National Registry 34% [24, 26]. Borzych-Duzalka et al. identified omental wrapping as a common cause of obstruction regardless of treatment duration, aligning with our findings that patients who underwent omentectomy experienced only two cases of obstruction [6]. In contrast to Gudsoorkar et al.’s report [27], which identified the fallopian tube as a frequent cause of catheter malfunction, this study found that males had a 2.14 hazard ratio of revision (*p* = 0.012) in both the acute and chronic PD groups.

Interestingly, highly experienced surgeons were associated with a tenfold increase in revision rates, likely due to their handling of more complex cases with a higher risk of failure. At UMC-HE, left-sided catheter implantation, intended to reserve the right side for future transplantation, did not correlate with a higher revision hazard. However, in children under 12 months of age, right-side implantation proved protective against complications. Although this approach contrasts with current guidelines, we advocate for right-sided implantation when it facilitates more secure and effective catheter placement, especially in patients unlikely to require future transplantation.

Centralization of PD patients in high-volume centres has reduced reported peritonitis incidence [15, 19]. Despite this, peritonitis, along with tunnel and exit site infections, remains the most common complications, with incidence rates varying significantly between centres [22, 28]. Comparable with Radtke et al.’s findings, this study observed a 24% incidence of first peritonitis, with age and chronic PD identified as risk factors. While the omentum itself has immunologic functions [21, 22], our results suggest that neither omentectomy nor implantations side is associated with higher peritonitis rates. Indeed, other centres reported significantly lower rates of peritonitis in patients who underwent omentectomy [23, 25].

In our analysis, the peritonitis incidence increased by 7% with each additional year of age, likely due to increased catheter manipulation and mobility in older patients. Experiencing peritonitis within the first 3 months doubled the risk of subsequent episodes. We observed a peritonitis rate of 0.06 per patient-year, which is lower than published rates in the USA, UK, and Australia (0.25 and 0.36 per patient-year, respectively) [13, 16, 21]. A possible interpretation of this is that during the first period of PD, patients are selected on the basis of their adherence to procedures and prescriptions takes place, and only those with greater compliance continue to receive treatment.

An interesting subgroup with an increased incidence of complications emerged: children with eGFR between 15 and 29 ml/min/1.73 m^2^. Although this group does not require PD, selected patients may benefit from a short PD period when they cannot maintain euvolemia—for example, due to severe tissue oedema, or prolonged surgical stress [15, 33]. The management of these patients remains a challenge, as their overall survival is negatively affected by complications [29]. However, initiating a short period of PD remains a common practice after cardiac surgery, and individualized assessment result in a lower communication rate.

Hernias are common complication of, affecting up to 25% of patients within 2 years of starting PD, due to increased intra-abdominal pressure during dialysate dwells [27, 28]. This study observed a 14% incidence of hernias and 2% incidence of hydroceles in male toddlers and young children. The incidence was 8.85-fold greater in kidney disease patients in this study. The correlation between renal function and malnutrition in patients with renal failure has been well described [30, 31] and is mostly addressed with gastrostomies [32]. Gastrostomy tube insertion at or after PD catheter placement was associated with peritonitis, as leakage of PD fluid or stomach contents has been reported in 7.9% of cases [4, 25, 30]. Therefore, PD catheter placement should be done prior to or simultaneously with TC placement [30].

Comparing complication rates between the acute and chronic PD groups, peritonitis and revision were less common in the acute PD group (aHR = 0.09, *p* = 0.001; aHR = 0.35, *p* = 0.055). The patients necessitating acute PD are in a fragile situation, and may necessitate switching to other renal replacement therapies. These factors require further research as they could influence the overall complication risk in acute PD.

While acknowledging the limitations of a single-centre retrospective study and the limited ability of the Poisson analysis to identify all risk factors, our study’s strengths include a long follow-up period and a large patient cohort. The significant number of newborns, infants, and toddlers treated with strict adherence to surgical technique guidelines enhances the validity of our results. In the future, combined prospective studies between specialized PD centres will strengthen the evidence, and enable earlier recognition of high-risk patients in order to reduce complication risks.

## Conclusion

This study reaffirms neonates and infants as a high-risk group and highlights male patients with kidney disease as particularly vulnerable. Children undergoing PD for non-cardiac or renal issues also face an elevated risk of complications. The risk of peritonitis in our cohort increased by 7% per year of age, with a twofold risk of recurrence if the first episode occurred within 3 months after implantation. These findings underscore the importance of close monitoring, particularly in the early post-implantation period. Factors such as the side of implantation and prior abdominal surgeries may also influence outcomes warranting careful surgical planning. Clinicians should remain alert to hernia development, especially in male infants and toddlers, and carefully assess the timing of gastrostomy tube placement to minimize complications related to nutritional support.

## Supplementary Information

Below is the link to the electronic supplementary material.

Supplementary file1 (DOCX 18 KB)

Supplementary file2 (PNG 69 KB)

Supplementary file3 (DOCX 31 KB)

## Data Availability

No datasets were generated or analysed during the current study.

## Abbreviations

GFR: Glomerular filtration rate
PD: Peritoneal dialysis
TC: Tenckhoff catheter
AKI: Acute kidney injury
ESRD: End-stage renal disease
ISPD: International Society for Peritoneal Dialysis
ECMO: Extracorporeal membrane oxygenation
UMC-HE: University Medical Centre Hamburg, Eppendorf
