# Supplementary material for: Surgical risk stratification and outcome analysis of Tenckhoff catheter implantations in paediatric patients: a single-centre experience
Source: Eur J Pediatr. 2025 Feb 4;184(2):172. doi: 10.1007/s00431-025-06006-x (PMC11794337; doi:10.1007/s00431-025-06006-x)
Supplement: Supplementary file 1 — Supplementary file1 (DOCX 18 KB) [file 431_2025_6006_MOESM1_ESM.docx]

Catheter survival analysis-Πες μου αν θες να προστεθουν ή να αφαιρεθουν μεταβλητες

| Factor |  | N (%) or Median (IQR) | HR with 95% CI | aHR with 95% CI |
| --- | --- | --- | --- | --- |
|  | Chronic | 110 (44.7) | Ref. | Ref. |
|  | Acute | 136 (55.3) | 2.52 (1.94-3.29, p<0.001) | 3.61 (2.20-5.94, p<0.001) |
| Sex | Female | 116 (47.2) | Ref. | Ref. |
|  | Male | 130 (52.8) | 0.97 (0.75-1.25, p=0.802) | 1.04 (0.72-1.49, p=0.847) |
| Age Group | Newborn | 53 (21.5) | Ref. | Ref. |
|  | Infant | 32 (13.0) | 1.01 (0.64-1.59, p=0.969) | 1.02 (0.54-1.95, p=0.941) |
|  | Toddler | 80 (32.5) | 1.18 (0.83-1.67, p=0.363) | 0.53 (0.27-1.03, p=0.063) |
|  | School child | 44 (17.9) | 0.72 (0.48-1.08, p=0.117) | 0.53 (0.27-1.05, p=0.067) |
|  | Post adolescent, Adolescent, Adults | 37 (15.0) | 0.67 (0.44-1.02, p=0.063) | 0.49 (0.22-1.11, p=0.086) |
| Diseases Group | Heart disease | 29 (11.8) | Ref. | Ref. |
|  | Kidney disease | 209 (85.0) | 0.50 (0.34-0.74, p=0.001) | 1.85 (0.86-3.98, p=0.114) |
|  | Other* | 8 (3.3) | 0.64 (0.29-1.40, p=0.261) | 3.56 (1.10-11.50, p=0.034) |
| Omentectomy | No | 180 (76.3) | Ref. | Ref. |
|  | Yes | 56 (23.7) | 0.93 (0.69-1.25, p=0.625) | 0.70 (0.45-1.08, p=0.110) |
| Side of Implantation | Left | 122 (61.6) | Ref. | Ref. |
|  | Right | 71 (35.9) | 1.03 (0.77-1.39, p=0.831) | 1.11 (0.76-1.60, p=0.596) |
|  | Other | 5 (2.5) | 1.54 (0.63-3.79, p=0.347) | 2.13 (0.67-6.75, p=0.200) |
| experience | Low experience | 41 (20.4) | Ref. | Ref. |
|  | Moderate experience | 64 (31.8) | 0.67 (0.45-1.00, p=0.052) | 0.69 (0.40-1.18, p=0.178) |
|  | High experience | 96 (47.8) | 0.67 (0.47-0.98, p=0.036) | 0.71 (0.42-1.19, p=0.198) |
| BMI | 5th percentile to less than the 85th percentile | 102 (57.6) | Ref. | Ref. |
|  | Less than the 5th percentile | 50 (28.2) | 1.66 (1.18-2.35, p=0.004) | 1.13 (0.73-1.74, p=0.590) |
|  | 85th percentile to less than the 95th percentile | 12 (6.8) | 0.85 (0.46-1.56, p=0.600) | 0.72 (0.36-1.44, p=0.358) |
|  | 95th percentile or greater | 13 (7.3) | 1.37 (0.77-2.45, p=0.287) | 1.39 (0.68-2.83, p=0.372) |
| Creatinine | Mean (SD) | 5.1 (8.1) | 1.00 (0.97-1.04, p=0.917) | 1.05 (0.97-1.15, p=0.236) |
| GFR | 90 | 2 (1.1) | 1.50 (0.37-6.08, p=0.570) | - |
|  | 60-89 | 2 (1.1) | 3.87 (0.94-15.88, p=0.060) | 4.18 (0.85-20.49, p=0.078) |
|  | 30-59 | 8 (4.4) | 0.94 (0.43-2.05, p=0.871) | 1.29 (0.48-3.45, p=0.609) |
|  | 15-29 | 19 (10.5) | 1.89 (1.17-3.08, p=0.010) | 2.19 (1.16-4.14, p=0.016) |
|  | <15 | 150 (82.9) | Ref. | Ref. |
| No of Operations | 1 | 204 (82.9) | Ref. | Ref. |
|  | 2 | 30 (12.2) | 1.12 (0.76-1.65, p=0.559) | 1.44 (0.85-2.45, p=0.177) |
|  | 3 | 11 (4.5) | 1.48 (0.80-2.72, p=0.213) | 2.08 (0.78-5.57, p=0.143) |
|  | 5 | 1 (0.4) | 1.09 (0.15-7.82, p=0.929) | 1.06 (0.13-8.99, p=0.956) |
| *Bowel disease, metabolic disease, trauma (+1pts kidney&heart disease)  HR: Hazard Ratio; aHR: adjusted Hazard Ratio; CI: Confidence Interval | | | | |
