# Supplementary figures and images for: Surgical risk stratification and outcome analysis of Tenckhoff catheter implantations in paediatric patients: a single-centre experience
Source: Eur J Pediatr. 2025 Feb 4;184(2):172. doi: 10.1007/s00431-025-06006-x (PMC11794337; doi:10.1007/s00431-025-06006-x)

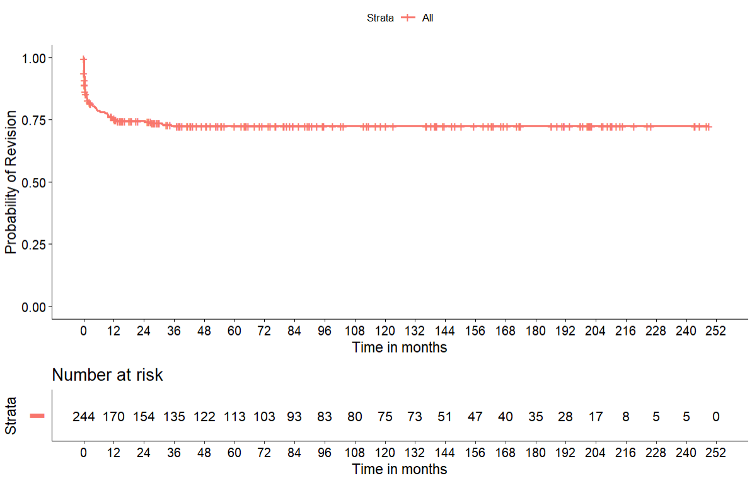

Supplement: Supplementary file 2 — Supplementary file2 (PNG 69 KB) [file 431_2025_6006_MOESM2_ESM.png]
