# Supplementary material for: Surgical risk stratification and outcome analysis of Tenckhoff catheter implantations in paediatric patients: a single-centre experience
Source: Eur J Pediatr. 2025 Feb 4;184(2):172. doi: 10.1007/s00431-025-06006-x (PMC11794337; doi:10.1007/s00431-025-06006-x)
Supplement: Supplementary file 3 — Supplementary file3 (DOCX 31 KB) [file 431_2025_6006_MOESM3_ESM.docx]

| Factor  Table 1. Overall survival in participants aged up to 12 months | | all | HR with 95% CI | aHR with 95% CI |
| --- | --- | --- | --- | --- |
| Sex | Female | 30 (35.3) | Ref. | Ref. |
|  | Male | 55 (64.7) | 1.08 (0.51-2.32, p=0.835) | 0.91 (0.23-3.64, p=0.893) |
| Age (days) | Mean (SD) | 64.6 (100.6) | 1.00 (1.00-1.00, p=0.609) | 1.00 (0.99-1.01, p=0.906) |
| Diseases Group | Heart disease | 25 (29.4) | Ref. | Ref. |
|  | Kidney disease | 56 (65.9) | 0.43 (0.21-0.91, p=0.027) | 0.47 (0.07-3.06, p=0.431) |
|  | Other | 4 (4.7) | 1.13 (0.26-5.03, p=0.868) | 5.91 (0.68-51.25, p=0.107) |
| Gestations Age | Full Term (>37) | 41 (51.9) | Ref. | Ref. |
|  | Extremely PT (<28) | 2 (2.5) | 1.89 (0.25-14.60, p=0.540) | - (p=0.999) |
|  | Very PT (>28 - <32) | 5 (6.3) | 3.83 (1.22-12.03, p=0.021) | 16.22 (1.77-148.83, p=0.014) |
|  | Moderately PT (>32 - <34) | 8 (10.1) | 2.98 (0.96-9.30, p=0.060) | 11.67 (0.56-242.88, p=0.113) |
|  | Late PT (>34 - <37) | 23 (29.1) | 1.21 (0.48-3.08, p=0.689) | 2.72 (0.46-15.87, p=0.267) |
| Omentectomy | No | 63 (76.8) | Ref. | Ref. |
|  | Yes | 19 (23.2) | 0.60 (0.23-1.56, p=0.294) | 0.22 (0.03-1.96, p=0.176) |
| Side of Implantation | Left | 48 (61.5) | Ref. | Ref. |
|  | Right | 29 (37.2) | 0.61 (0.27-1.38, p=0.234) | 0.16 (0.03-0.89, p=0.037) |
|  | Other | 1 (1.3) | 6.75 (0.85-53.94, p=0.072) | 0.86 (0.02-47.29, p=0.941) |
| Experience | Low experience | 8 (10.3) | Ref. | Ref. |
|  | Moderate experience | 24 (30.8) | 2.46 (0.55-10.88, p=0.237) | - |
|  | High experience | 46 (59.0) | 1.16 (0.26-5.11, p=0.843) | - |
| BMI | 5th percentile to less than the 85th percentile | 36 (53.7) | - | - |
|  | Less than the 5th percentile | 25 (37.3) | 0.72 (0.27-1.95, p=0.521) | 0.62 (0.17-2.20, p=0.457) |
|  | 85th percentile to less than the 95th percentile | 3 (4.5) | - (p=0.998) | - (p=0.998) |
|  | 95th percentile or greater | 3 (4.5) | 3.47 (0.73-16.37, p=0.117) | 1.02 (0.04-25.92, p=0.993) |
| Creatinine | Mean (SD) | 2.6 (1.6) | 0.74 (0.58-0.96, p=0.023) | 0.82 (0.47-1.44, p=0.497) |
| GFR | <15 | 53 (74.6) | Ref. | Ref. |
|  | 90 | 1 (1.4) | - (p=0.998) |  |
|  | 60-89 | 2 (2.8) | 3.75 (0.48-29.05, p=0.206) | 0.33 (0.02-4.99, p=0.426) |
|  | 30-59 | 6 (8.5) | 1.86 (0.53-6.47, p=0.332) | 1.81 (0.24-13.44, p=0.560) |
|  | 15-29 | 9 (12.7) | 0.96 (0.22-4.25, p=0.961) | 0.32 (0.04-2.89, p=0.311) |
| Number of Peritonitis | 0 | 62 (72.9) | Ref. | Ref. |
|  | 1 | 20 (23.5) | 0.28 (0.08-0.92, p=0.036) | - |
|  | 2 | 3 (3.5) | - (p=0.997) | - |
| Revision | No | 52 (61.2) | Ref. | Ref. |
|  | Yes | 33 (38.8) | 0.82 (0.39-1.72, p=0.601) | - |

Table 2. Peritonitis, time to event analysis in patients aged up to 12 months

|  | | All | HR with 95% CI | aHR with 95% CI |
| --- | --- | --- | --- | --- |
| Sex | Female | 30 (35.3) | Ref. | Ref. |
|  | Male | 55 (64.7) | 1.05 (0.45-2.48, p=0.909) | 2.08 (0.37-11.84, p=0.408) |
| Age (days) | Mean (SD) | 64.6 (100.6) | 1.00 (1.00-1.00, p=0.854) | 1.00 (0.99-1.01, p=0.875) |
| Diseases | Heart disease | 25 (29.4) | Ref. | Ref. |
|  | Kidney disease | 56 (65.9) | 2.02 (0.68-5.97, p=0.204) | 0.98 (0.12-7.96, p=0.985) |
|  | Other | 4 (4.7) | 1.35 (0.15-12.10, p=0.787) | - (p=0.999) |
| BMI | 5th percentile to less than the 85th percentile | 36 (53.7) | Ref. | Ref. |
|  | Less than the 5th percentile | 25 (37.3) | 1.64 (0.65-4.13, p=0.297) | 1.05 (0.28-3.90, p=0.946) |
|  | 85th percentile to less than the 95th percentile | 3 (4.5) | 1.21 (0.15-9.55, p=0.857) | 1.46 (0.11-18.78, p=0.771) |
|  | 95th percentile or greater | 3 (4.5) | 1.38 (0.17-10.87, p=0.762) | 1.26 (0.05-29.65, p=0.887) |
| Creatinine | Mean (SD) | 2.6 (1.6) | 1.20 (0.96-1.50, p=0.115) | 1.43 (0.95-2.16, p=0.084) |
| GFR | <15 | 53 (74.6) | Ref. | Ref. |
|  | 90 | 1 (1.4) | - (p=0.998) | - (p=0.999) |
|  | 60-89 | 2 (2.8) | - (p=0.998) | - (p=0.999) |
|  | 30-59 | 6 (8.5) | 0.58 (0.08-4.37, p=0.597) | 2.69 (0.11-64.68, p=0.542) |
|  | 15-29 | 9 (12.7) | 2.67 (0.97-7.31, p=0.056) | 10.70 (1.34-85.20, p=0.025) |
| Gestations Age | Full Term (>37) | 41 (51.9) | Ref. | Ref. |
| (weeks) | Extremely PT (<28) | 2 (2.5) | - (p=0.997) | - (p=0.999) |
|  | Very PT (>28 - <32) | 5 (6.3) | 0.94 (0.12-7.53, p=0.955) | 2.07 (0.18-24.46, p=0.562) |
|  | Moderately PT (>32 - <34) | 8 (10.1) | 4.31 (1.40-13.24, p=0.011) | 15.44 (2.50-95.42, p=0.003) |
|  | Late PT (>34 - <37) | 23 (29.1) | 1.81 (0.65-4.99, p=0.254) | 11.04 (0.78-155.46, p=0.075) |
| Gastric Tube | No | 62 (72.9) | Ref. | Ref. |
|  | Yes | 23 (27.1) | 2.92 (1.29-6.63, p=0.010) | 0.51 (0.07-3.85, p=0.511) |
| *Bowel disease, metabolic disease, trauma (+1pts kidney&heart disease)  HR: Hazard Ratio; aHR: adjusted Hazard Ratio; CI: Confidence Interval | | | | |

Table 3. Hernia, time to event analysis (all patients)

|  | | all | HR with 95% CI | aHR with 95% CI |
| --- | --- | --- | --- | --- |
| Sex | Female | 116 (47.2) | Ref. | Ref. |
|  | Male | 130 (52.8) | 3.60 (1.56-8.29, p=0.003) | 1.78 (0.70-4.54, p=0.224) |
| Age Group | Newborn | 53 (21.5) | Ref. | Ref. |
|  | Infant | 32 (13.0) | 0.38 (0.13-1.13, p=0.082) | 0.17 (0.02-1.38, p=0.097) |
|  | Toddler | 80 (32.5) | 0.28 (0.12-0.66, p=0.004) | 0.25 (0.08-0.74, p=0.012) |
|  | School child | 44 (17.9) | 0.25 (0.08-0.75, p=0.013) | 0.07 (0.01-0.43, p=0.004) |
|  | Post adolescent, Adolescent, Adults | 37 (15.0) | 0.08 (0.01-0.58, p=0.013) | 0.04 (0.00-0.54, p=0.015) |
| Disease | Heart disease | 29 (11.8) | Ref. | Ref. |
|  | Kidney disease | 209 (85.0) | 2.34 (0.56-9.80, p=0.243) | 8.85 (0.72-108.38, p=0.088) |
|  | Other | 8 (3.3) | - (p=0.997) | - (p=0.999) |
| Omentectomy | No | 180 (76.3) | Ref. | Ref. |
|  | Yes | 56 (23.7) | 1.28 (0.57-2.88, p=0.543) | 1.28 (0.49-3.32, p=0.616) |
| Side of Implantation | Left | 122 (61.6) | Ref. | Ref. |
|  | Right | 71 (35.9) | 1.20 (0.58-2.49, p=0.626) | 0.74 (0.31-1.74, p=0.484) |
|  | Other | 5 (2.5) | - (p=0.996) | - (p=0.999) |
| experience | Low experience | 41 (20.4) | Ref. | Ref. |
|  | Moderate experience | 64 (31.8) | 1.47 (0.39-5.54, p=0.572) | 1.49 (0.28-7.97, p=0.642) |
|  | High experience | 96 (47.8) | 2.55 (0.75-8.63, p=0.132) | 1.91 (0.40-9.20, p=0.420) |
| BMI | 5th percentile to less than the 85th percentile | 102 (57.6) | Ref. | Ref. |
|  | Less than the 5th percentile | 50 (28.2) | 0.74 (0.31-1.78, p=0.509) | 0.67 (0.25-1.79, p=0.424) |
|  | 85th percentile to less than the 95th percentile | 12 (6.8) | 1.33 (0.39-4.50, p=0.651) | 0.98 (0.25-3.78, p=0.976) |
|  | 95th percentile or greater | 13 (7.3) | 0.41 (0.05-3.06, p=0.384) | - (p=0.998) |
| Creatinine | Mean (SD) | 5.1 (8.1) | 0.93 (0.82-1.06, p=0.278) | 1.09 (0.89-1.32, p=0.403) |
| GFR | <15 | 150 (82.9) | Ref. | Ref. |
|  | 90 | 2 (1.1) | 3.25 (0.44-24.00, p=0.248) | - |
|  | 60-89 | 2 (1.1) | - (p=0.998) | - (p=0.999) |
|  | 30-59 | 8 (4.4) | 0.68 (0.09-5.03, p=0.708) | 3.92 (0.29-53.85, p=0.306) |
|  | 15-29 | 19 (10.5) | 0.57 (0.13-2.38, p=0.438) | 0.84 (0.17-4.17, p=0.828) |
| *Bowel disease, metabolic disease, trauma (+1pts kidney&heart disease)  HR: Hazard Ratio; aHR: adjusted Hazard Ratio; CI: Confidence Interval | | | | |

Table 4. Hernia, time to event analysis in patients with kidney disease

|  | | all | HR (univariable) | HR (multivariable) |
| --- | --- | --- | --- | --- |
| Sex | Female | 100 (47.8) | Ref. | Ref. |
|  | Male | 109 (52.2) | 3.46 (1.49-8.03, p=0.004) | 1.70 (0.66-4.40, p=0.273) |
| Age Group | Newborn | 40 (19.1) | Ref. | Ref. |
|  | Infant | 17 (8.1) | 0.43 (0.12-1.49, p=0.184) | 0.20 (0.02-1.66, p=0.136) |
|  | Toddler | 75 (35.9) | 0.23 (0.10-0.53, p=0.001) | 0.22 (0.07-0.70, p=0.010) |
|  | School child | 42 (20.1) | 0.19 (0.06-0.58, p=0.004) | 0.07 (0.01-0.41, p=0.003) |
|  | Post adolescent, Adolescent, Adults | 35 (16.7) | 0.06 (0.01-0.46, p=0.007) | 0.04 (0.00-0.51, p=0.014) |
| Omentectomy | No | 151 (75.1) | Ref. | Ref. |
|  | Yes | 50 (24.9) | 1.09 (0.47-2.57, p=0.838) | 1.25 (0.48-3.28, p=0.651) |
| Side of Implantation | Left | 103 (62.8) | Ref. | Ref. |
|  | Right | 58 (35.4) | 1.20 (0.56-2.57, p=0.634) | 0.84 (0.35-2.02, p=0.696) |
|  | Other | 3 (1.8) | 0.00 (0.00-Inf, p=0.997) | 0.00 (0.00-Inf, p=0.999) |
| Experience | Low experience | 38 (22.8) | Ref. | Ref. |
|  | Moderate experience | 45 (26.9) | 1.71 (0.44-6.63, p=0.438) | 1.09 (0.18-6.39, p=0.927) |
|  | High experience | 84 (50.3) | 2.56 (0.75-8.69, p=0.132) | 1.61 (0.32-8.02, p=0.562) |
| BMI | 5th percentile to less than the 85th percentile | 89 (61.0) | Ref. | Ref. |
|  | Less than the 5th percentile | 35 (24.0) | 0.81 (0.32-2.04, p=0.655) | 0.62 (0.22-1.78, p=0.377) |
|  | 85th percentile to less than the 95th percentile | 11 (7.5) | 1.25 (0.37-4.25, p=0.718) | 1.06 (0.27-4.12, p=0.935) |
|  | 95th percentile or greater | 11 (7.5) | 0.42 (0.06-3.15, p=0.400) | 0.00 (0.00-Inf, p=0.997) |
| Creatinine | Mean (SD) | 5.7 (8.7) | 0.85 (0.72-1.00, p=0.048) | 1.10 (0.90-1.34, p=0.347) |
| GFR | <15 | 137 (91.3) | Ref. | Ref. |
|  | 90 | 1 (0.7) | 8.26 (1.09-62.39, p=0.041) | - |
|  | 60-89 | 0 (0.0) | - | - |
|  | 30-59 | 1 (0.7) | 7.77 (1.03-58.47, p=0.046) | 12.18 (0.96-155.04, p=0.054) |
|  | 15-29 | 11 (7.3) | 0.45 (0.06-3.33, p=0.436) | 0.42 (0.05-3.60, p=0.430) |
| *Bowel disease, metabolic disease, trauma (+1pts kidney&heart disease)  HR: Hazard Ratio; aHR: adjusted Hazard Ratio; CI: Confidence Interval | | | | |

Table 5. Incidence of peritonitis

|  | | Incidence Rate Ratios with 95%CI | Sig. | Adjusted Incidence Rate Ratios with 95%CI | Sig. |
| --- | --- | --- | --- | --- | --- |
| Age | | 1.07 (1.03 – 1.10) | <0.001 | 1.07 (1.00 – 1.15) | 0.045 |
| Sex | Male | 1.31 (0.87 – 2.00) | 0.193 | 1.75 (0.87 -3.50) | 0.116 |
|  | Female | Ref. |  | Ref. |  |
| Disease | Heart disease | Ref. |  | Ref. |  |
|  | Kidney disease | 1.70 (0.85 – 4.06) | 0.175 | 0.54 (015 -2.02) | 0.364 |
|  | Other* | 0.52 (0.03 – 2.91) | 0.538 | - | 0.992 |
| Omentectomy | Yes | 0.74 (0.42 – 1.22) | 0.260 | 0.74 (0.33 -1.64) | 0.458 |
|  | No | Ref. |  | Ref. |  |
| Creatinine | | 1.01 (0.99 – 1.02) | 0.249 | 1.07 (0.95 – 1.20) | 0.282 |
| BMI | 5th percentile to less than the 85th percentile | Ref. |  | Ref. |  |
|  | Less than the 5th percentile | 0.61 (0.32 – 1.07) | 0.102 | 0.62 (0.23 – 1.72) | 0.362 |
|  | 85th percentile to less than the 95th percentile | 0.54 (0.13 – 1.48) | 0.736 | 1.34 (0.39 – 4.63) | 0.639 |
|  | 95th percentile or greater | 0.83 (0.29 – 1.91) | 0.701 | 0.42 (0.05 – 3.29) | 0.410 |
| GRF | >=90 | - | 0.992 | - |  |
|  | 60-89 | - | 0.992 | - |  |
|  | 30-59 | 0.56 (0.14 – 2.28) | 0.419 | - |  |
|  | 15-29 | 0.59 (0.24 – 1.46) | 0.254 | 2.25 (0.28 – 18.37) | 0.449 |
|  | <15 | Ref |  | Ref. |  |
| Gastric tubes | Yes | 1.49 (0.80 – 2.55) | 0.175 | 1.94 (0.76 – 4.96) | 0.167 |
|  | No | Ref. |  | Ref. |  |
| First peritonitis 3months | Yes | 7.29 (4.86 – 10.99) | <0.001 | 2.53 (1.15 – 5.55) | 0.021 |
|  | No | Ref. |  | Ref. |  |
| *Bowel disease, metabolic disease, trauma (+1pts kidney&heart disease)  IRR: Incidence Rate Ratios; aIRR: Adjusted Incidence Rate Ratios; CI: Confidence Interval | | | | | |
